# The Effect of SARS-CoV-2 Vaccination on HIV Viral Load in Patients Under Bictegravir/Tenofovir Alafenamide/Emtricitabine Therapy: A Retrospective Observational Study

**DOI:** 10.3390/healthcare13080926

**Published:** 2025-04-17

**Authors:** Giuseppe Pipitone, Giacomo Ciusa, Stefano Agrenzano, Francesco Di Lorenzo, Caterina Sagnelli, Antonio Cascio, Chiara Iaria, TheBICivico StudyGroup

**Affiliations:** 1 Infectious Disease Unit, ARNAS Civico-Di Cristina, 90100 Palermo, Italy; 2 Department of Mental Health and Public Medicine, Section of Infectious Diseases, University of Campania “Luigi Vanvitelli”, 80131 Naples, Italy; 3 Infectious Disease Unit, University Hospital P. Giaccone, 90127 Palermo, Italy

**Keywords:** SARS-CoV-2 infection, people living with HIV, PLWH, HIV infection, blip, bictegravir

## Abstract

**Background:** The aim of our study is to evaluate the impact of SARS-CoV-2 vaccination on HIV viremia in patients treated under bictegravir-based therapy. **Methods:** We conducted a retrospective observational study in a tertiary hospital, analyzing data from 152 patients treated with BIC/TAF/FTC between 2020 and 2022. Patients were divided into two groups: “vaccinated” (110/152) and “unvaccinated” (42/152) against SARS-CoV-2. The outcomes considered were the presence of “blips” (detectable viremia ≥ 20 copies/mL), “rebound” (viremia ≥ 50 copies/mL), and virological failures. **Results:** A lower incidence of blips in the “vaccinated” group compared to the “unvaccinated” group (9.1% vs. 28.6%, *p* = 0.002), and a reduced risk of blips in the vaccinated group (OR 3.8, 95% CI 1.4–9.8) were noticed. The rebound rate was lower in the vaccinated group compared to non-vaccinated, with a statistically significant difference (respectively, 2.7% vs. 11.9%, *p* = 0.037). **Conclusions:** our data suggest that SARS-CoV-2 vaccination may stimulate an immune response that enhances CD4+ and CD8+ cell function, contributing to a reduction in the number of blips and maintaining good viro-immunological control in patients with HIV, supporting the importance of vaccination in this population.

## 1. Introduction

The coronavirus disease 2019 (COVID-19) pandemic has represented a heavy commitment for healthcare professionals and a challenge for the healthcare system. During this period, many hospitals in Italy had to reduce the services provided and many infectious disease clinics dedicated to the treatment of human immunodeficiency virus (HIV) infection had to interrupt their service. Others, while keeping the service active, have noted an increase in the number of missed visits, a reduction in new HIV diagnoses, and an increase in the incidence of hospitalizations among people living with HIV (PLWH) [1].

Although cases of transiently detectable HIV viremia after the Severe Acute Respiratory Syndrome COronaVirus-2 (SARS-CoV-2) vaccine in a patient on antiretroviral therapy (ART) have been described by Bozzi G et al. [2], the association between vaccination and viremic blips is not unambiguously defined [3].

Among ART regimens, bictegravir, tenofovir alafenamide, and emtricitabine (BIC/TAF/FTC) have been widely used due to their efficacy and safety. This ART has a strong virological response and low resistance rates [4,5,6]. However, the effects of SARS-CoV-2 vaccination on ART remain unclear in terms of HIV viral load and immune response. Several studies have raised concerns about potential HIV viremia “blips” in vaccinated patients, maybe due to vaccine-induced immune activation [7,8]. Blips, defined as transient episodes of detectable viremia (HIV-RNA ≥ 20 copies/mL) below the threshold for virological failure, have been associated with increased immune activation and a heightened risk of HIV reservoir activation. They are often temporary and clinically insignificant, affecting around 10–15% of HIV patients, and they have been associated with immune system changes or, in some cases, virological rebound in patients with an unstable immune profile [9,10].

Blips and rebound may pose clinical concerns in PLWH on ART, and they could be an early indicator of virological failure or immune uncontrol. Some authors suggest that vaccine-induced immune stimulation could cause a short-term increase in HIV-RNA levels [11,12,13]. This poses questions about the interaction between SARS-CoV-2 vaccination and HIV management, and whether vaccination might increase the risk of blips or even virological failure among patients on specific ART regimens. So, periodically monitoring HIV-RNA is mandatory.

Despite concerns, recent studies indicate that vaccination may enhance immune responses in PLWH, promoting CD4+ and CD8+ T-cell activity for both HIV and SARS-CoV-2 control [14,15,16]. Vaccination could therefore play a role in sustaining immunological health by cross-activating immune responses that could help HIV management in vaccinated patients on ART.

During the pandemic, we noticed some viral blips in stably viro-suppressed patients in BIC/TAF/FTC therapy after vaccination, and we tried to analyze if there could be an association between the events.

## 2. Materials and Methods

**Figure 1:**
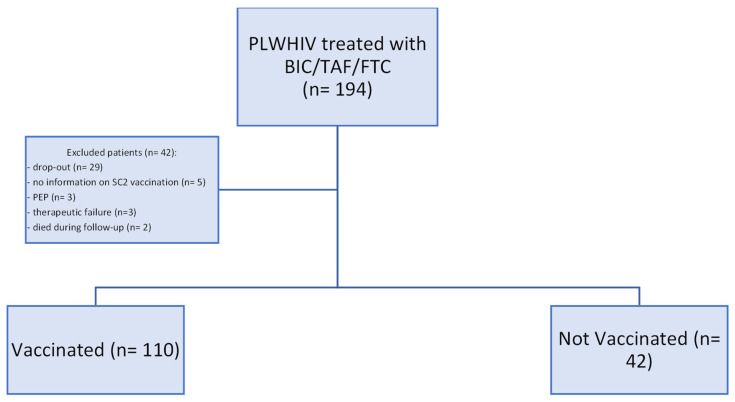
Patients’ enrollment flow chart. PLWHIV: people living with HIV. HIV: Human immunodeficiency virus. BIC: bictegravir. TAF: Tenofovir alafenamide. FTC: Emtricitabine. SC2: SARS-CoV-2. PEP: post-exposure vaccination.

In this observational retrospective study we analyzed data collected via electronic medical records of 194 patients prescribed BIC/TAF/FTC between 1 January 2020 and 31 December 2022. The study was carried out by collecting data from electronic records in the period between 1 January 2020 and 1 March 2023. Patients were included if they had a BIC/TAF/FTC regimen for an HIV infection, they were divided among “Vaccinated” and “Unvaccinated” groups based on their SARS-CoV-2 vaccination status, and they were evaluated during the follow-up period (Figure 1). We defined a “blip” as the presence of detectable viremia (VR) with HIV-RNA > 20 copies/mL and “rebound” as a viremia with HIV-RNA ≥ 50 copies/mL.

Age, sex, starting day of BIC/TAF/FTC, immunovirological data as CD4, CD4/CD8 values, the presence of a blip or rebound, and any COVID-19 vaccinations were collected. Virological failure was defined when a patient had at least two viremias > 200 copies/mL in at least two consecutive detections. Quantitative variables are shown as median and interquartile range (IQR) 25–75%, with a 95% confidence interval (CI).

Quantitative variables were analyzed by the ANOVA test of variance with Levine’s test, 95% CI, and an alfa = 0.05. Qualitative variables were analyzed using Fisher’s exact test or the Chi-square test. A logistic regression analysis was performed for confounding factors. SPSS Statistics^®^ software (v. 29) was used for the statistical analysis.

## 3. Results

Among 194 patients who were prescribed BIC/TAF/FTC during the observation period, 42 patients were excluded for the following reasons: the prescription was carried out as post-exposure prophylaxis (*n* = 3), drop-out patients (*n* = 29), died during the follow-up period (*n* = 2), no information was available for SARS-CoV-2 vaccination (*n* = 5), 1 patient has never reduced his viral load to zero since being on ART, and 2 patients do not regularly take therapy. These last 3 patients were therefore defined as therapeutic failures (Figure 1).

The statistical analysis was conducted among 152 patients (Table 1). Patients were divided in two groups: vaccinated and not vaccinated.

The overall population was predominantly composed of males (105/152, 69.1%), without statistically significant differences between the two groups (*p* = 0.699), and the median age was 54 years (IQR 43–60, 95% CI) without differences between the two groups (*p* = 0.180).

Patients had a good immunological status with a median CD4+ T lymphocyte count of 594 cells/mL (IQR 415–791, 95% CI) and a CD4+/CD8+ ratio of 0.9 (IQR 0.6–1.3, 95% CI), and no statistically significant difference between the two groups was found (*p* = 0.709 and *p* = 0.166, respectively). Data on CD4+ and CD4+/CD8+ ratio were missing for one patient. Patients have been on BIC/TAF/FTC combination therapy for a median time of 23.5 months (IQR 14–28.75, 95% CI). A longer duration of therapy was observed in the vaccinated group compared to the non-vaccinated group (respectively, 25 vs. 17.5 months, *p* = 0.009). Patients who presented a blip were 22/152 (14.5%), with a lower rate of blips in the vaccinated group compared to the non-vaccinated group (respectively, 9.1% vs. 28.6%, *p* = 0.002). We observed rebound overall in 8/152 (5.3%), with a lower rate in the vaccinated group compared to non-vaccinated (2.7% vs. 11.9%, *p* = 0.037). Among the 22 patients who had presented with a blip, the blip “event” occurred with a median of 3.5 months (IQR 2–7.5, 95% CI). To evaluate the risk of a blip we performed a logistic regression analysis for confounding factors (Table 2).

The risk of a blip in the non-vaccinated group is estimated in OR 3.78 (95% CI, 1.46–9.78). Finally, among vaccinated patients, no association was found between a blip and the type of vaccine (mRNA or SARS-CoV-2 spike protein vaccines) nor the number of vaccine doses (Table 3).

## 4. Discussion

During the pandemic, no virological failure was detected among patients included in the analysis, and the incidence of blips appears comparable to that detected by other real-life studies preceding this period, as observed by Bouchard et al. [4,17,18].

In our study, we observed a lower incidence of blips and rebound among vaccinated compared to non-vaccinated patients. Furthermore, logistic regression analysis confirmed a higher risk of a blip among non-vaccinated patients. The observation period between vaccination and hypothetical blips was considered from the day after vaccination up to 12 months after. Vogel et al. [5] proposed extending the observation period for post-vaccination immunological reactions to 12 weeks; in our study, 5/10 (50%) of the blips occurred within this time frame, while 3/10 (30%) occurred 4–6 months after vaccination, and 2/10 (20%) occurred 12 months after vaccination.

Viral blips are a frequent phenomenon among HIV patients on ART and are linked to an increased risk of virological failure; however, the causes of such blips could be for multiple reasons, including the presence of a large viral reservoir and the clonal expansion of CD4+ T lymphocytes [7]. Furthermore, an immune stimulus could represent another cause of transiently detectable viremia [8].

Previously, some authors have reported the possible correlation between vaccination and a transient increase in viremia due to immunological hyperactivation [8,9]. The correlation between anti-SARS-CoV-2 vaccination, well-tolerated in PLWH [19], and viral blips is still the subject of debate [3,10,11].

Given the higher rate of blips in the non-vaccinated group compared to the vaccinated group, our hypothesis is diametrically opposed to the previously mentioned authors; the vaccine’s immune hyperstimulation (or a natural infection), lasting several months, confers a cross-lymphocyte activation in HIV infected cells. The hypothesis is based on literature evidence of a sustained lymphocyte response on SARS-CoV-2 vaccination, lasting up to 2–3 months or up to 1 year after vaccination for neutralizing antibodies [12,20,21]. This immunogenic stimulation could induce enhanced CD4+ and CD8+ functions, as confirmed by other studies [13]. SARS-CoV-2 vaccination does not induce changes in HIV viremia [14], and it is demonstrated that it could be associated with a lower incidence of blips by increasing the number and function of CD4+ and CD8+ cells [15].

These findings may suggest that vaccine-induced immune activation can stimulate the immune system in PLWH by enhancing T-cell functionality (expecially CD8+) and may lead to HIV viral control. [16].

Our findings suggest that the brief period of immune activation (due to vaccination) may enhance immune responses, potentially reducing the size of latent HIV reservoirs. This observation could be a positive implication of vaccination for PLWH [22]. Furthermore, vaccine-induced immune activation is transient [23], it does not increase the risk of rebound [14,24] and may contribute to stimulating a robust T-cell response [25,26].

Logistic regression analysis demonstrated that non-vaccinated individuals had a nearly four-fold higher risk of a blip compared to vaccinated patients. This supports the hypothesis that vaccination may promote T-cell functions with durable T-cell responses lasting over a year in PLWH, potentially reducing the likelihood of viral rebound or immune instability [27], converse to what was observed in PLWH after SARS-CoV-2 infection [25,28,29,30,31].

Finally, as some authors stated [32], both PLWH and those who are HIV negative experienced a robust T-cell immune response after the second SARS-CoV-2 vaccine dose, and the majority of our patients had two or three doses, which is compatible with a good immunity response.

The results of our study are confirmed by data from the literature and reiterate how an INSTI-based regimen could maintain good viro-immunological control in HIV patients. Furthermore, SARS-CoV-2 vaccination could induce a wide immunity response that may enhance the lymphocyte response to HIV infection as well as reducing the risk of developing severe forms of COVID-19 [33,34].

Our study has the following limitations: it is a retrospective observational study, and the observational design may pose a selection bias and limit our ability to establish a definitive causation between vaccination and reduced viral blips. Additionally, our cohort included only patients on BIC/TAF/FTC therapy, so we should be careful when generalizing to all PLWH patients on ART. Future studies should investigate these effects across diverse ART regimens and patient demographics. As for selection bias, we may hypothesize that vaccinated patients may have a higher awareness about their health and may be more likely involved in the medical system. Finally, the low number of patients, as well as the one-drug regimen, may affect the final results.

## 5. Conclusions

Our study show that SARS-CoV-2 vaccination may the improve immune response to HIV and lead to virological control in PLWH, particularly in those receiving BIC/TAF/FTC therapy. By promoting T-cell activation, vaccination could reduce the risk of transient viremia (blip or rebound), and it could support viral suppression. The results of our study highlight the importance of vaccination in PLWH, for both protection against SARS-CoV-2 infection and probable immunological control of HIV viremia.

Further research is needed to understand the long-term effects of SARS-CoV-2 vaccination on immune system response and virological control in other ART regimens and specific HIV populations (i.e., HIV naïve or patients with virological failure).

Finally, future research should highlight post-vaccination T-cell dynamics and HIV reservoir size, helping to clarify the exact pathways by which SARS-CoV-2 vaccines may benefit HIV viral control [8,14,35,36].

In conclusion, our study supports SARS-CoV-2 vaccination in PLWH and emphasizes the potential dual benefit of vaccination: protection against SARS-CoV-2 infection and a strengthened immune response in PLWH on ART. These findings may improve health policies for PLWH and help this vulnerable population in achieving durable viral suppression and an enhanced quality of life.

## Figures and Tables

**Table 1:** Patients’ characteristics are described. Age, sex, lymphocyte CD4+ values, CD4+/CD8+ ratio, the time difference between vaccination and a blip, the time difference from the start of therapy with bictegravir and the blip were collected. BIC = Bictegravir/tenofovir alafenamide/emtricitabine. Data are presented as median and IQR 25–75%, or number and percentage (%). Delta time vaccination–blip: time difference between vaccination and blip observation. Delta time BIC–blip: time difference between starting BIC therapy and blip observation. ^1^ data point is missing for one patient.

	Overall (*n* = 152)	Vaccinated (*n* = 110)	Non-Vaccinated (*n* = 42)	*p*-Value
Age (years)	54 (43–60)	55 (44–60)	51 (39.5–60.25)	0.180
Male Sex–*n* (%)	105/152 (69.1%)	75/110 (68.2%)	30/42 (71.4%)	0.699
CD4+ (cells/uL) ^1^	594 (415–791)	540 (415–774.5) ^1^	677.5 (421.75–838.25)	0.709
CD4+/CD8+ ratio ^1^	0.9 (0.6–1.3)	0.9 (0.6–1.3) ^1^	1 (0.7–1.3)	0.166
BIC therapy (months)	23.5 (14–28.75)	25 (16–30)	17.5 (9–27.5)	**0.009**
Blip	22/152 (14.5%)	10/110 (9.1%)	12/42(28.6%)	**0.002**
Rebound	8/152 (5.3%)	3/110 (2.7%)	5/42(11.9%)	**0.037**
Delta time vaccination–blip (months)	-	3.5 (2–7.5)	-	-
Delta time BIC–blip (months)	13 (9–16)	9.5 (5.75–15.25)	14 (9–19)	0.569

**Table 2:** Logistic regression analysis evaluating the risk of blip among patients. M: male.

Risk of Blip	OR	95% CI	*p*-Value
Age	0.985	0.950–1.021	0.407
Sex (M)	0.623	0.205–1.895	0.404
CD4+	1.001	0.999–1.003	0.510
CD4+/CD8+	0.603	0.170–2.132	0.432
Not vaccinated	**3.778**	**1.460**–**9.775**	**0.006**

**Table 3:** Patients’ vaccination characteristics among the vaccinated group.

	Vaccinated (*n* = 110)	Blip (*n* = 10)	Non Blip (*n* = 100)	*p*-Value
Type of vaccine-*n* (%)				
mRNA	106/110 (96.5%)	10/10 (100%)	96/100 (96%)	1
Tot. n. of vaccines-*n* (%)				
1	2/110 (1.8%)	0/10	2/100	0.646
2	16/110 (14.5%)	3/10	13/100	
3	72/110 (65.5%)	6/10	66/100	
4	19/110 (17.3%)	1/10	18/100	
5	1/110 (0.9%)	0/10	1/100	
N. of vaccination on blip				
Second dose	-	4/10	-	-
Third dose	-	6/10	-	-

## Data Availability

The original contributions presented in this study are included in the article/Supplementary Materials. Further inquiries can be directed to the corresponding author(s).

## Supplementary Materials

The following supporting information can be downloaded at: https://www.mdpi.com/article/10.3390/healthcare13080926/s1, STROBE Statement—Checklist of items that should be included in reports of case-control studies.
